# Circulating miR-489 as a potential new biomarker for idiopathic dilated cardiomyopathy

**DOI:** 10.20407/fmj.2020-001

**Published:** 2020-07-14

**Authors:** Tomoya Ishiguro, Mutsuharu Hayashi, Wakaya Fujiwara, Satoshi Okumura, Masataka Yoshinaga, Ryo Yamada, Sayano Ueda, Takehiro Ito, Yudai Niwa, Akane Miyazaki, Masahide Harada, Hiroyuki Naruse, Junnichi Ishii, Yukio Ozaki, Hideo Izawa

**Affiliations:** 1 Department of Cardiology, Fujita Health University Bantane Hospital, Nagoya, Aichi, Japan; 2 Department of Cardiology, Fujita Health University, School of Medicine, Toyoake, Aichi, Japan

**Keywords:** MicroRNA, Biomarker, Microarray, Dilated cardiomyopathy (DCM)

## Abstract

**Objectives:**

MicroRNAs (miRNA) are functional RNAs that have emerged as pivotal gene expression regulators in cardiac disease. Although several cardiomyocyte miRNAs have been reported to play roles in heart failure progression among patients with idiopathic dilated cardiomyopathy (DCM), the role of circulating miRNAs has not yet been well-examined.

**Methods:**

After total RNA extraction from the peripheral blood samples of three control participants and six patients with DCM, miRNA profiling was performed using miRNA arrays. Based on the results of this initial screening, real-time polymerase chain reaction (RT-PCR) was used to perform a quantitative analysis of blood samples from a larger number of matched patients (DCM, *n*=20; controls, *n*=5). Finally, the correlations between specific miRNA expression levels and hemodynamic parameters were analyzed.

**Results:**

A primary screening of 2,565 miRNAs resulted in the identification of nine miRNA candidates. Quantitative RT-PCR results revealed significantly increased miR-489 expression levels in the DCM group. Moreover, there was a significant positive correlation between miR-489 expression level and left ventricular ejection fraction.

**Conclusions:**

Our results suggest that circulating miR-489 could be a potential noninvasive diagnostic biomarker for DCM. Additionally, the quantification of circulating miR-489 may have value as a potential prognostic marker for patients with DCM.

## Introduction

MicroRNAs (miRNAs), which are noncoding RNAs that are 21–25 nucleotides in length, are generally considered to act as intracellular endogenous RNAs that regulate post-translational gene expression.^1,2^ Dysregulation of intracellular miRNA expression has been detected in various diseases, including several cardiovascular disorders.^3,4^ Furthermore, recent studies have demonstrated that miRNAs are detectable and highly stable in circulating blood, and that these circulating miRNAs may serve as biomarkers for early disease detection and prognosis.^5^ Although the physiological roles and importance of circulating miRNAs are not yet well-understood, their utility and practicability as potential biomarkers for various diseases has attracted much attention, especially in cancer.^6,7^ In cardiovascular diseases, distinctive patterns of circulating miRNAs have been identified for myocardial infarction, coronary artery disease, heart failure (HF), type 2 diabetes mellitus, and hypertension. Several studies involving congestive HF have evaluated the relationship between miRNA expression profiles and underlying pathological conditions, including cardiac fibrosis, cardiac hypertrophy, ventricular remodeling, and cardiac failure.^8–11^ Our group has previously revealed associations between reduced catecholamine sensitivity and several miRNAs extracted from right ventricular myocardium biopsy specimens in patients with idiopathic dilated cardiomyopathy (DCM).^3^ Furthermore, other studies have reported upregulated miR-423-5p and miR-361-5p expression in the myocardium of patients with DCM.^12^ However, little is known about the relationship between circulating miRNAs and DCM. The present study aimed to profile the expression levels of circulating cardiac-associated miRNAs in patients with DCM and determine their utility as biomarkers for DCM.

## Methods

### Diagnosis of DCM

DCM was diagnosed on the basis of <50% left ventricular ejection fraction (LVEF), as determined by contrast ventriculography, in the absence of the following: >50% coronary artery stenosis, as determined by coronary angiography; arterial hypertension; valvular heart disease; sustained atrial fibrillation; implantation of any mechanical cardiac support devices; complications that influence cardiac function, such as diabetes mellitus; chronic kidney disease and peripheral artery disease; and cardiac muscle disease secondary to any systemic disease.^13^ After considering both the risks and values for patient prognosis, magnetic resonance imaging and endomyocardial biopsies were not used in this study. Patients with DCM who had a New York Heart Association functional class of I or II with normal sinus rhythm were enrolled in the present study.

### Study protocol

A two-step process was used to investigate miRNA profiles. In six patients with DCM and three age- and sex-matched healthy controls with normal LVEF and coronary perfusion, we first performed a screening study using the microarray analysis system. Serum miRNA samples were hybridized to 3D-Gene Human miRNA chips (ver. 21) containing 2,565 miRNAs (Toray Industries, Inc., Tokyo, Japan). In this screening study, we selected candidate miRNAs in descending order of *p*-value for further detailed analysis using Quantitative Real-time Polymerase Chain Reaction (qRT-PCR).

The qRT-PCR analysis of the candidate miRNAs was performed in a larger number of age- and sex-matched patients (DCM group, *n*=20; control group, *n*=5) using a PRISM-7900HT thermocycler (Applied Biosystems, Foster City, CA, USA). *Caenorhabditis elegans* miR-39 (cel-miR-39) was spiked to each sample as a control for the extraction and amplification steps, because it has been reported that there are few differences between individuals in expression levels in RT-PCR. The relative expressions were calculated using the comparative ΔCT method with spiked cel-miR-39 levels. DCM-specific miRNA was detected when the expression level was significantly higher than either miR-16 or miR-423-3p, which were used as the internal controls.^14^

Hemodynamic parameters were assessed using echocardiography and blood chemistry analysis. Echocardiography was used to measure LVEF (using Simpson’s method), left ventricular end-systolic dimension (LVDs), left ventricular end-diastolic dimension (LVDd), left atrium diameter, fractional shortening (%), interventricular septum thickness, left ventricular posterior wall thickness, E/A (peak early diastolic LV filling velocity/peak atrial filling velocity ratio), and E/E' (peak early diastolic LV filling velocity/myocardial relaxation ratio). Blood tests included serum creatinine, hemoglobin, and B type natriuretic peptide (BNP) measurements.

The study protocol was approved by the Ethics Review Committee of Fujita Health University, and written informed consent was provided by each patient at the time of registration.

### Statistical analysis

Variables are presented as the mean±standard deviation (SD), and qualitative data are presented as percentages. Patient characteristics were assessed using Student’s *t*-test and Fisher’s exact test, and the results were analyzed using Student’s *t*-test. Correlations were tested using Pearson’s correlation coefficient. To compare differences in serum miRNA expressions between the DCM and control groups, Mann–Whitney *U* tests were used. All statistical analyses were performed using SPSS version 11.0 (SPSS Japan, Inc., Tokyo, Japan). Covariates that were found to be significant (*p*<0.05) during univariate analyses were incorporated into the multivariate analyses. All reported *p*-values are two-tailed, and statistical significance was set at *p*<0.01 for the microarray analyses and *p*<0.05 for the qRT-PCR analyses.

## Results

### miRNA microarray

The miRNA microarray analysis was performed to identify DCM-associated differences in circulating miRNA profiles between six patients with DCM and three controls. The baseline clinical characteristics of the DCM and control groups are shown in Table 1. There were no significant differences between the two groups except for the left ventricular contraction and diameter values. Of the 2,565 miRNAs in the miRNA chips, there were significant differences between the two groups in the expression levels of nine miRNAs (Table 2).

### qRT-PCR analysis

Table 3 shows the clinical characteristics of the two groups that were used for the qRT-PCR analysis, which comprised 20 patients with DCM and five control participants. There were no significant differences in age and sex between the two groups. Echocardiographic measurements revealed that patients with DCM had significantly lower LVEF and larger LVDd and LVDs compared with the controls. The left ventricular thickness and other structural and functional parameters were comparable in the two groups. The heart rates and QRS intervals in electrocardiograms between the two groups were not significantly different. Blood examinations indicated that both groups had comparable serum levels of high-sensitivity C-reactive protein, cardiac troponin I, creatinine, hemoglobin, and BNP. The DCM patients were only treated with cardiac protective agents, such as beta-blockers and angiotensin-converting enzyme inhibitors, because they had no major complications that influenced cardiac function.

We further examined the expression levels of the nine miRNAs identified during the first miRNA microarray screening, as well as those of three miRNAs (miR-1, miR-134, miR-423-5p)^15–17^ whose relationships with cardiomyocyte degradation are well-established. The levels of five miRNAs (miR-3156, miR-4721, miR-4745, miR-4781, and miR-8052) were below the limit of detection for qRT-PCR. However, the expression level of miR-489 was significantly higher in the DCM group than in the control group when using miR-16 as the internal control (*p*=0.03), and it also had a higher tendency in the DCM group when miR-423-3p was used as the internal control (*p*=0.09). In contrast, the levels of the remaining miRNAs and the previously reported three miRNAs were comparable between the two groups (Table 4).

### Association between miR-489 expression and LVEF

**Figure 1:**
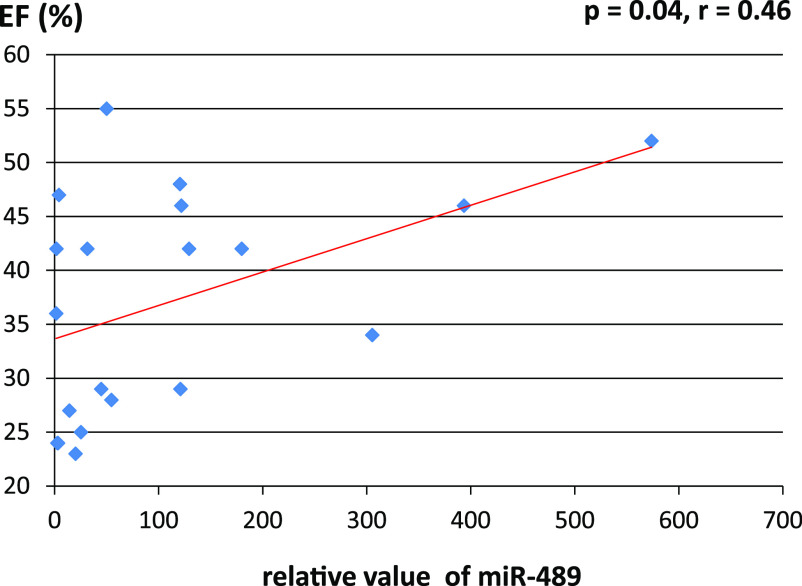
There was a significant positive association between LVEF and circulating miR-489 levels in patients with DCM (*p*=0.04, *r*=0.46). LVEF, left ventricular ejection fraction; DCM, idiopathic dilated cardiomyopathy.

When we analyzed the association between serum miR-489 levels and cardiac functional markers, there was a significant positive correlation between LVEF and miR-489 expression levels (*p*=0.04, *r*=0.46; Figure 1). There were no significant correlations between serum miR-489 levels and other hemodynamic parameters (data not shown).

## Discussion

DCM, which is transmitted by autosomal dominant inheritance and often results from mutations in multiple genes, is one of the most common causes of HF. Although DCM has been previously reported to have a poor prognosis,^18^ a large number of patients with DCM have had positive responses to pharmacological and mechanical therapies for left ventricular reverse remodeling, which confers a more favorable long-term prognosis. However, it remains challenging to identify patients with increased likelihoods of improvement after therapeutic optimization. Several miRNAs in human heart tissue have been suggested as valuable diagnostic and prognostic markers for HF.^19^ However, although numerous studies have been published, the reported impact of miRNAs in HF management remains variable.

The present study revealed that circulating miR-489 may be a potential noninvasive diagnostic biomarker for DCM, and that expression levels of circulating miR-489 may correlate with cardiomyocyte viability in patients with this disease. The expression levels of circulating miR-489 were significantly higher in patients with DCM than in controls in our study. Moreover, there was a positive correlation between circulating miR-489 levels and LVEF in patients with DCM. Given that DCM is a degenerative myocardial disease, a reduction in LVEF is mainly dependent on the degree of myocardial fibrogenesis. These results therefore indicate that myocardial degeneration upregulates circulating miR-489, whereas the progression of myocardial fibrogenesis decreases circulating miR-489. Thus, circulating miR-489 levels may serve as a valuable marker for detecting myocardial degeneration and identifying disease stage and prognosis in DCM.

Several investigations of the role of miR-489 have recently been published. Although recent reports have mainly focused on the role of miR-489 in suppressing tumor proliferation and metastasis,^20,21^ miR-489 dysregulation has also been reported in muscle-related disorders; it is downregulated in cardiac cachexia^22^ and upregulated in muscular dystrophy.^23^ These findings support the pivotal role of miR-489 in muscular turnover and degeneration, the depletion of fetal muscle cells, and fibrosis. Moreover, these observations with regard to skeletal muscle abnormalities are consistent with our present findings in cardiomyocytes.

We therefore suggest the utility of measuring miR-489 as a noninvasive diagnostic and prognostic biomarker for patients with DCM. Studies elucidating the importance of the miR-489 signaling axis in regulating cell proliferation and metastasis are ongoing. As such, we expect to further unravel the mechanisms behind the relationship between myocardial degeneration and miR-489 regulation, which would further benefit patients with DCM through prevalent miRNA screenings.

### Study limitations

Our study has several limitations. First, regarding the qRT-PCR analysis, multiplicity was not adequately considered. In this study, we conducted the microRNA array using miR-16 and miR-423-3p as the internal controls, based on a report by Sedigheh et al.^14^ However, this methodology is not standard, and reliable reference genes for quantifying miRNAs in serum samples have not yet been well-established.

Second, we revealed a significant positive correlation between miR-489 expression levels and LVEF. However, it is possible that the number of cases in our study was too small to conclude this association. Moreover, miR-489 expression levels were only significantly higher in the analysis using miR-16 as the internal control. More extensive studies are therefore needed to demonstrate the utility of miR-489 measurement as both a diagnostic and prognostic marker for DCM in the future.

## Figures and Tables

**Table1:** Baseline clinical characteristics of the DCM and control groups from the miRNA array

	DCM	Control	*p*-Value
	*n*=6	*n*=3	
Age (years)	53.5±14.8	53.7±7.37	0.49
Male (%)	50	67	0.65
LVEF (%)	30.3±7.23	63.3±2.31	<0.01
LVDd (mm)	69±15.3	42.7±1.15	0.02
LVDs (mm)	57±16.7	25.3±4.62	0.01
IVST (mm)	8.17±1.83	8±1	0.89
LVPWT (mm)	9.12±2.04	8±1	0.39
Heart rate (bpm)	66.2±5.08	74±8	0.11
QRS interval (msec)	97.9±5.81	91.8±2.11	0.12
BNP (pg/mL)	171.6±192.4	15.8±11.9	0.22
Hemoglobin (g/dL)	13.4±1.39	13.9±0.75	0.59
Creatinine (mg/dL)	0.71±0.16	0.77±0.18	0.65

Data are presented as the mean±SD. DCM, idiopathic dilated cardiomyopathy; LVEF, left ventricular ejection fraction; LVDd, left ventricular diastolic diameter; LVDs, left ventricular systolic diameter; IVST; interventricular septal thickness, LVPWT; left ventricular posterior wall thickness; BNP, brain natriuretic peptide.

**Table2:** Altered miRNA expression in blood samples from patients with idiopathic dilated cardiomyopathy (DCM)

	*p*-Value	Ratio (DCM/control)
miR-489	0.009	1.7
miR-496	0.005	0.56
miR-3156	0.008	0.56
miR-4721	0.001	0.66
miR-4745	0.002	0.69
miR-4781	0.005	0.6
miR-5010	0.007	0.73
miR-5088	0.008	1.38
miR-8052	0.001	0.68

Each *p*-value was calculated using a paired *t*-test.

**Table3:** Baseline clinical characteristics of the DCM and control groups from the RT-PCR experiments

	DCM	Control	*p*-Value
	*n*=20	*n*=5	
Age (years)	57.4±15.3	53±6	0.54
Male (%)	65	60	0.84
LVEF (%)	37.1±10.3	62.8±1.92	<0.01
LVDd (mm)	61.2±12.9	42.6±0.89	<0.01
LVDs (mm)	49.4±14.6	26.8±3.9	<0.01
IVST (mm)	8.2±2.6	9.3±3.1	0.65
LVPWT (mm)	8.1±2.8	8.9±2.8	0.54
Heart rate (bpm)	71.4±15.6	64.6±4.8	0.24
QRS interval (msec)	98.8±7.86	92.4±2.86	0.18
BNP (pg/mL)	117.4±148.8	15.68±10.2	0.15
Hemoglobin (g/dL)	13.6±1.34	13.1±1.5	0.49
Creatinine (mg/dL)	0.99±0.79	0.78±0.13	0.56

Data are presented as the mean±SD. DCM, idiopathic dilated cardiomyopathy; LVEF, left ventricular ejection fraction; LVDd, left ventricular diastolic diameter; LVDs, left ventricular systolic diameter; IVST; interventricular septal thickness, LVPWT; left ventricular posterior wall thickness, BNP, brain natriuretic peptide.

**Table4:** Quantitative comparison of candidate miRNAs and previous reported myocardium miRNAs between DCM and control patients

Internal control	miR-16			miR-423-3p		
	DCM	Control	*p*-Value	DCM	Control	*p*-Value
	*n*=20	*n*=5		*n*=20	*n*=5	
miR-489	109.9±151.8	36.4±39.6	0.03	133.9±328.3	32±37.4	0.09
miR-496	6.45±11.3	1.36±1.61	0.06	2.32±3.15	1.07±1.14	0.11
miR-5010	40.3±47.9	27.2±43.8	0.33	15.2±16.4	19.8±31.5	0.41
miR-5088	222.7±268.5	105.1±136.8	0.1	229.4±552.5	91.6±123.6	0.16
miR-1	2.17±2.81	2.75±5.01	0.31	1.34±1.54	2.2±1.51	0.15
miR-134	77±113	33.5±49	0.11	96.3±245	31±49.3	0.14
miR-423-5p	15.1±16.7	11.6±13.8	0.33	5.61±4.3	9.46±10.2	0.22

Relative miRNA expressions were calculated using the comparative ΔCT method with spiked *C. elegans* miR-39. Data are presented as the mean±SD. DCM, idiopathic dilated cardiomyopathy.

## References

[1] Ambros V. The functions of animal microRNAs. Nature 2004; 431: 350–355. 15372042 10.1038/nature02871

[2] Kaneda R, Fukuda K. MicroRNA is a new diagnostic and therapeutic target for cardiovascular disease and regenerative medicine. Circ J 2009; 73: 1397–1398. 10.1253/circj.cj-09-0435 19628922

[3] Funahashi H, Izawa H, Hirashiki A, Cheng XW, Inden Y, Nomura M, Murohara T. Altered microRNA expression associated with reduced catecholamine sensitivity in patients with chronic heart failure. J Cardiol 2011; 57: 338–344. 21367584 10.1016/j.jjcc.2011.01.009

[4] Lu HQ, Liang C, He ZQ, Wu ZG. Circulating miR-214 is associated with the severity of coronary artery disease. J Geriatr Cardiol 2013; 10: 34–38. 23610572 10.3969/j.issn.1671-5411.2013.01.007 PMC3627710

[5] Mitchell PS, Parkin RK, Kroh EM, et al. Circulating microRNAs as stable blood-based markers for cancer detection. Proc Natl Acad Sci U S A 2008; 105: 10513–10518. 18663219 10.1073/pnas.0804549105 PMC2492472

[6] Chen X, Ba Y, Ma L, et al. Characterization of microRNAs in serum: a novel class of biomarkers for diagnosis of cancer and other diseases. Cell Res 2008; 18: 997–1006. 18766170 10.1038/cr.2008.282

[7] Kosaka N, Iguchi H, Ochiya T. Circulating microRNA in body fluid: a new potential biomarker for cancer diagnosis and prognosis. Cancer Sci 2010; 101: 2087–2092. 20624164 10.1111/j.1349-7006.2010.01650.x PMC11159200

[8] Sayed D, Hong C, Chen IY, Lypowy J, Abdellatif M. MicroRNAs play an essential role in the development of cardiac hypertrophy. Circ Res 2007; 100: 416–424. 17234972 10.1161/01.RES.0000257913.42552.23

[9] Villar AV, Garcia R, Merino D, Llano M, Cobo M, Montalvo C, Martín-Durán R, Hurlé MA, Nistal JF. Myocardial and circulating levels of microRNA-21 reflect left ventricular fibrosis in aortic stenosis patients. Int J Cardiol 2013; 167: 2875–2881. 22882958 10.1016/j.ijcard.2012.07.021

[10] Wang J, Huang W, Xu R, Nie Y, Cao X, Meng J, Xu X, Hu S, Zheng Z. MicroRNA-24 regulates cardiac fibrosis after myocardial infarction. J Cell Mol Med 2012; 16: 2150–2160. 22260784 10.1111/j.1582-4934.2012.01523.x PMC3822985

[11] Montgomery RL, Hullinger TG, Semus HM, Dickinson BA, Seto AG, Lynch JM, Stack C, Latimer PA, Olson EN, van Rooij E. Therapeutic inhibition of miR-208a improves cardiac function and survival during heart failure. Circulation 2011; 124: 1537–1547. 21900086 10.1161/CIRCULATIONAHA.111.030932 PMC3353551

[12] Fan KL, Zhang HF, Shen J, Zhang Q, Li XL. Circulating microRNAs levels in Chinese heart failure patients caused by dilated cardiomyopathy. Indian Heart J 2013; 65: 12–16. 23438607 10.1016/j.ihj.2012.12.022 PMC3860780

[13] Richardson P, McKenna W, Bristow M, Maisch B, Mautner B, O’Connell J, Olsen E, Thiene G, Goodwin J, Gyarfas I, Martin I, Nordet P. Report of the 1995 World Health Organization/International Society and Federation of Cardiology Task Force on the Definition and Classification of cardiomyopathies. Circulation 1996; 93: 841–842. 8598070 10.1161/01.cir.93.5.841

[14] Sedigheh G, Mehdi S, Shahriar K, Mohammad RS, Nassim G, Mahmood T, Mohammad RN, Seyed JM. Identification of reliable refernce Genes for quantification of microRNAs in serum of sulfur mustard-exposed veterans. Cell Journal 2015; 17: 494–501. 26464821 10.22074/cellj.2015.9 PMC4601870

[15] Ai J, Zhang R, Li Y, Pu J, Lu Y, Jiao J, Li Z, Wang R, Wang L, Li Q, Wang N, Shan H, Li Z, Yang B. Circulating microRNA-1 as a potential novel biomarker for acute myocardial infarction. Biochem Biophys Res Commun 2010; 391: 73–77. 19896465 10.1016/j.bbrc.2009.11.005

[16] Tijsen AJ, Creemers EE, Moerland PD, de Windt LJ, van der Wal AC, Kok WE, Pinto YM. MiR423-5p as a circulating biomarker for heart failure. Circ Res 2010; 106: 1035–1039. 20185794 10.1161/CIRCRESAHA.110.218297

[17] Xiao J, Jing ZC, Ellinor PT, et al. MicroRNA-134 as a potential plasma biomarker for the diagnosis of acute pulmonary embolism. J Transl Med 2011; 9: 159. 21943159 10.1186/1479-5876-9-159 PMC3189884

[18] Scrutinio D, Napoli V, Passantino A, Ricci A, Lagioia R, Rizzon P. Low-dose dobutamine responsiveness in idiopathic dilated cardiomyopathy: relation to exercise capacity and clinical outcome. Eur Heart J 2000; 21: 927–934. 10806017 10.1053/euhj.1999.1937

[19] Melman YF, Shah R, Das S. MicroRNAs in heart failure: is the picture becoming less miRky? Circ Heart Fail 2014; 7: 203–214. 24449811 10.1161/CIRCHEARTFAILURE.113.000266

[20] Pashaei E, Ahmady M, Ozen M, Aydin N. Meta-analysis of miRNA expression profiles for prostate cancer recurrence following radical prostatectomy. PLoS One 2017; 12: e0179543. 28651018 10.1371/journal.pone.0179543 PMC5484492

[21] Li J, Qu W, Jiang Y, Jiang Y, Sun Y, Cheng Y, Zou T, Du S. miR-489 suppresses proliferation and invasion of human bladder cancer cells. Oncol Res 2016; 24: 391–398. 28281959 10.3727/096504016X14666990347518 PMC7838638

[22] Moraes LN, Fernandez GJ, Vechetti-Junior IJ, Freire PP, Souza RWA, Villacis RAR, Rogatto SR, Reis PP, Dal-Pai-Silva M, Carvalho RF. Integration of miRNA and mRNA expression profiles reveals microRNA-regulated networks during muscle wasting in cardiac cachexia. Sci Rep 2017; 7: 6998. 28765595 10.1038/s41598-017-07236-2 PMC5539204

[23] Sylvius N, Bonne G, Straatman K, Reddy T, Gant TW, Shackleton S. MicroRNA expression profiling in patients with lamin A/C-associated muscular dystrophy. FASEB J 2011; 25: 3966–3978. 21840938 10.1096/fj.11-182915

